# Efficacy Evaluation of Neurofeedback-Based Anxiety Relief

**DOI:** 10.3389/fnins.2021.758068

**Published:** 2021-10-28

**Authors:** Chao Chen, Xiaolin Xiao, Abdelkader Nasreddine Belkacem, Lin Lu, Xin Wang, Weibo Yi, Penghai Li, Changming Wang, Sha Sha, Xixi Zhao, Dong Ming

**Affiliations:** ^1^Academy of Medical Engineering and Translational Medicine, Tianjin University, Tianjin, China; ^2^Key Laboratory of Complex System Control Theory and Application, Tianjin University of Technology, Tianjin, China; ^3^Department of Computer and Network Engineering, College of Information Technology, United Arab Emirates University, Al Ain, United Arab Emirates; ^4^Zhonghuan Information College, Tianjin University of Technology, Tianjin, China; ^5^Beijing Machine and Equipment Institute, Beijing, China; ^6^Department of Neurosurgery, Xuanwu Hospital, Capital Medical University, Beijing, China; ^7^Brain-Inspired Intelligence and Clinical Translational Research Center, Xuanwu Hospital, Capital Medical University, Beijing, China; ^8^Beijing Key Laboratory of Mental Disorders, Beijing Anding Hospital, Capital Medical University, Beijing, China

**Keywords:** neurofeedback, anxiety disorder, EEG signal, anxiety assessment, efficacy evaluation

## Abstract

Anxiety disorder is a mental illness that involves extreme fear or worry, which can alter the balance of chemicals in the brain. This change and evaluation of anxiety state are accompanied by a comprehensive treatment procedure. It is well-known that the treatment of anxiety is chiefly based on psychotherapy and drug therapy, and there is no objective standard evaluation. In this paper, the proposed method focuses on examining neural changes to explore the effect of mindfulness regulation in accordance with neurofeedback in patients with anxiety. We designed a closed neurofeedback experiment that includes three stages to adjust the psychological state of the subjects. A total of 34 subjects, 17 with anxiety disorder and 17 healthy, participated in this experiment. Through the three stages of the experiment, electroencephalography (EEG) resting state signal and mindfulness-based EEG signal were recorded. Power spectral density was selected as the evaluation index through the regulation of neurofeedback mindfulness, and repeated analysis of variance (ANOVA) method was used for statistical analysis. The findings of this study reveal that the proposed method has a positive effect on both types of subjects. After mindfulness adjustment, the power map exhibited an upward trend. The increase in the average power of gamma wave indicates the relief of anxiety. The enhancement of the wave power represents an improvement in the subjects’ mindfulness ability. At the same time, the results of ANOVA showed that *P* < 0.05, i.e., the difference was significant. From the aspect of neurophysiological signals, we objectively evaluated the ability of our experiment to relieve anxiety. The neurofeedback mindfulness regulation can effect on the brain activity pattern of anxiety disorder patients.

## Introduction

Anxiety is an emotional response to a potential future threat or danger that, depending on intensity and duration, can cause symptoms of negative emotional, physical, behavioral, and cognitive components. While “normal” anxiety is adaptive to make the body alert and prepare it for potential threats, it is considered pathological when it becomes maladaptive, permanent, and out of control. Furthermore, it is associated with serious social and occupational harm, other comorbidities, and an increased risk of suicide (Nepon et al., 2010). The classification of anxiety disorders has a long history (Crocq, 2015). According to the International Statistical Classification of Diseases and Related Health Problems (ICD-10), anxiety disorders are classified into generalized anxiety disorder, phobias, social anxiety disorder, post-traumatic stress disorder (PTSD), panic disorder with/without agoraphobia, and obsessive–compulsive disorder (OCD) (Kogan et al., 2016; Reed et al., 2019). Vulnerability to the development of anxiety disorders (Otowa et al., 2016; Gottschalk and Domschke, 2018) usually begins in childhood or adolescence (Kalin, 2017) and becomes a chronic condition that persists into adulthood (Bandelow and Michaelis, 2015; Craske et al., 2017). In the western world, the lifetime prevalence of these diseases in the general population is about 20–30%, making it the most common neuropsychiatric disorder, with women more susceptible than men (Revicki et al., 2012; Remes et al., 2016; GBD 2017 DALYs and Hale Collaborators, 2018). In summary, anxiety disorders impose a staggering burden on public health and global economy, highlighting the dire need to develop a more comprehensive understanding of the underlying mechanisms (Mokdad et al., 2018).

Current treatment options are mainly on psychotherapy and medication, which has proven effective in anxiety disorders (Carpenter et al., 2018). Psychological therapy is time-consuming and requires extensive training of therapists. Non-compliance, non-response, or incomplete response, and relapse are still major issues in patients receiving treatment (Taylor et al., 2012; Roy-Byrne, 2015). Currently available drug treatments for anxiety include selective serotonin reuptake inhibitors and serotonin and norepinephrine reuptake inhibitors, and benzodiazepines are most suitable for short-term and adjuvant antianxiety therapy. Traditional Chinese medicine injections and oral contraceptives are effective, but tolerance-related problems restrict their usage. It is encouraging that new mechanical compounds targeting glutamate, neuropeptides, and the endocannabinoid system are also being developed; however, there is insufficient information regarding the role of the glutamate system in the pathogenesis and persistence of anxiety disorders (Bandelow et al., 2016), and cannabis itself increases the risk of anxiety attacks (Grunberg et al., 2015; Mammen et al., 2018). In addition to the compounds covered in the current review, other potentially promising areas for future research include components of the neurotrophic signaling, renin–angiotensin, acetylcholine, and even the opioid system (Morrison and Ressler, 2014). In conclusion, there is still an urgent need to develop novel methods to treat anxiety disorders and related diseases (Griebel and Holmes, 2013). In a recent review, Markiewcz (2017) showed that neurofeedback is effective in many psychiatric disorders that affect psychological variables such as stress and anxiety. To avoid the side effects of drugs, from the perspective of anxiety-reducing technology (Pintado and Llamazares, 2014), neurofeedback therapy is a promising new method with stable and lasting therapeutic effects no side effects.

A number of studies in the extant literature have affirmed that in the treatment of anxiety disorders, neurofeedback focuses on the central nervous system and the brain (Fovet et al., 2015) to improve neuroregulation and stability. Among them, the regulation of brain activity can affect behavioral changes (Marzbani et al., 2016; Van der Kolk et al., 2016). Neurofeedback uses computer technology to train patients to improve poorly regulated brain wave patterns (Micoulaud-Franchi et al., 2015). Current imaging modes of neurofeedback include real-time magnetic resonance imaging (RT-MRI), functional near-infrared spectroscopy (fNIRS), and electroencephalography (EEG). For example, Lori-Ann et al. (2013) explored frontal lobe asymmetry using fNIRS. To assess the prefrontal asymmetry of female college students with the highest and lowest percentile scores in the high and low anxiety groups on social challenge tasks *in vivo*, the results showed that the high anxiety group exhibited a non-significant trend toward greater right frontal activity than the low anxiety group but only to assess the prefrontal cortex. For example, Morgenroth et al. (2020) assigned 32 participants with high trait anxiety to either an experimental group to undergo RT-MRI or a control group to receive a false feedback. The results showed that RT-fMRI neurofeedback training led to a reduction in anxiety levels and the feasibility of altering activation in the wider network. However, there was no group difference in Stroop’s task performance. In studies such as Sachs et al. (2004) using quantitative EEG to compare participants with a healthy control group in a state of rest and alertness (the participants would sound an alarm if drowsiness occurred), Sachs and his colleagues observed population differences in beta frequencies in the frontal lobe and right central region. Although no statistical analysis of hemispheric data was performed, the beta acceleration appeared to be predominantly in the right hemisphere. Subjects with high or low trait anxiety used alpha feedback to increase and decrease their EEG alpha activity. Changes in alpha were strongly associated with changes in anxiety but only in subjects with a high level of anxiety (for whom anxiety decreased linearly with an increase in alpha and increased linearly with an increase in alpha inhibition). These results suggest that long-term alpha feedback training (at least 5 h) may be helpful in anxiety management.

This study is based on an evaluation of the efficacy of an anxious state classification described in Chao et al. (2021), where in EEG signals were used to study neural changes, and the results showed that the support vector machine classifier was able to classify and recognize two psychological states (anxiety and no anxiety) using power spectral density as a model. In this paper, we design a neurofeedback system based on the alpha band oscillation (frequency power) of EEG signals. Subjects with anxiety disorders and healthy subjects were recruited to participate in the experiment where EEG signals were recorded and analyzed. The findings revealed that the activity of alpha, theta, and gamma waves of anxious subjects increased significantly. After the adjustment of mindfulness, the observation power graph showed an increasing trend. At the same time, the analysis of variance (ANOVA) showed that *P* < 0.05, i.e., the difference was significant, and the anxiety symptoms of the subjects could be relieved from the perspective of neurophysiology.

## Materials and Methods

### Subjects

In this study, a total of 34 subjects, 17 with anxiety disorder (37 ± 7.61 years old) and 17 healthy (24.41 ± 1.49 years old) participated in this experiment. All the subjects had normal hearing and never received mindfulness recording therapy or training. These anxiety disorders were judged by professional psychiatrists from Beijing Anding Hospital affiliated with Capital Medical University. All procedures performed in studies involving human participants were in accordance with the ethical standards of ethics committee of Beijing Anding Hospital, Capital Medical University (ZYLX201607). Healthy subjects are graduate students. Prior to the experiment, the subjects were instructed to read and sign the informed consent form and detailed personal information. The subjects were classified as healthy or anxious. The anxiety targets were pure anxiety patients. All the subjects participated in this experiment in a psychiatric hospital with consent. The selection criteria were decided by professional psychiatrists to appraise the subjects’ eligibility to participate in this experiment.

### Experiment Paradigm and Data Recording

**FIGURE 1:**
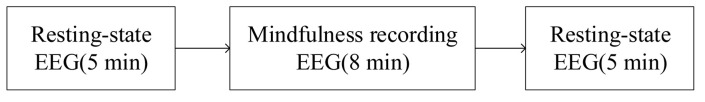
Experimental paradigm of the proposed affective brain–computer interface.

The subjects were asked to sit in a chair facing the desktop computer. As shown in Figure 1, the example includes three stages. In the first stage, the subjects were asked to remain emotionally stable for 5 min. The mindfulness recording was played in the second stage; the subjects followed the mindfulness (Schwartz et al., 2003; Sever et al., 2003) recording and gave an 8 min voice prompt to adjust their mental state. Finally, the subjects continued to return to a static state for 5 min. In this experiment, all subjects were required to keep their eyes open. In three small experimental phases, they needed to complete a self-assessment of anxiety. Each subject was required to fill in a visual analog scale comprising a scale axes marked with numbers 0–10. The number from 0 to 4 are defined as non-anxious, from 5 to 7 as moderate anxiety, and 8 to 10 as severe anxiety.

**FIGURE 2:**
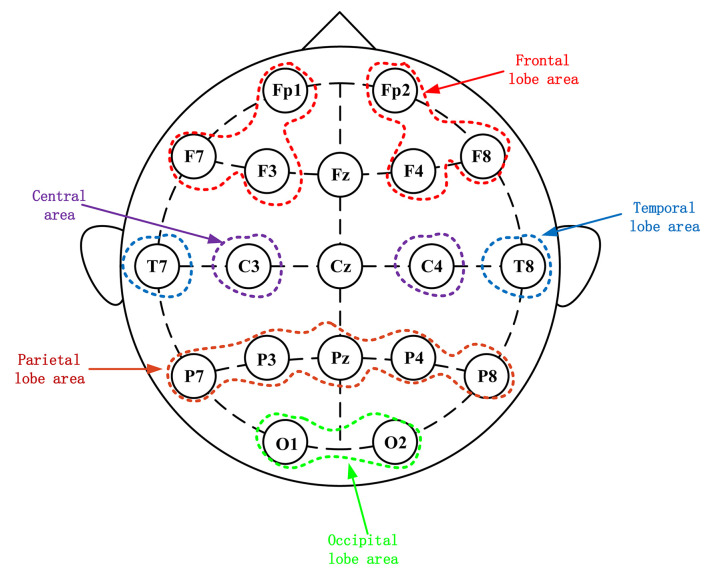
Electroencephalography electrode placement based on the international 10–20 system. Scalp potential and reference point distribution of left and right frontal lobes.

During the experiment, a 32-channel EEG signal was recorded from the subjects’ scalp (Brain Products, Germany). According to the International 10–20 system, the EEG signals were recorded through 19 electrodes, namely Fp1, Fp2, F7, F3, F4, F8, T7, C3, C4, T8, P7, P3, Pz, P4, P8, O1, O2, Fz, and Cz as shown in Figure 2. The electrode Cz were chosen as reference electrodes. During data recording, the impedance of each electrode was kept below 5 KΩ. The EEG data were collected at a sampling rate of 500 Hz.

**FIGURE 3:**
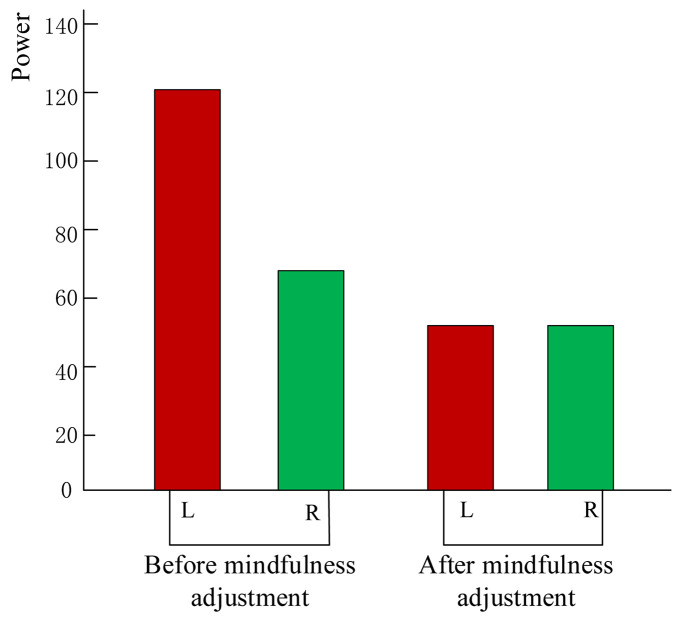
Subject neurofeedback presentation using energy change diagram of left and right frontal lobes.

Throughout this experiment, the alpha band power of the electrodes in the left and right frontal lobes (Harmon-Jones and Allen, 1997) was calculated in real time and displayed as feedback to establish a neurofeedback system. The energy of the signal is shown as red and green bars to depict the energy asymmetry in the frontal lobe. The red and green bar graphs represent the energy values of the alpha wave on the left and right sides of the frontal lobe, respectively. Subjects can see the changes in the visual bar and try to adjust their mental state during mindfulness training. As shown in Figure 3, prior to the mindfulness training, the left and right strengths are different, while subsequent to the training, the strength difference of some subjects decreased.

### Electroencephalography Data Processing

Because there are many noise artifacts in EEG signals, such as electrocardiogram (ECG), electromyography (EMG), and power frequency interference, it is necessary to preprocess the original EEG signal to obtain a relatively pure brain signal. In this study, independent component analysis (Arnaud, 2004) was used to eliminate eye movement artifacts. Our preprocessed EEG data took 4 s as the step size, calculated the power spectral density (Ahani et al., 2014; Cai et al., 2018), and obtained the alpha, theta, and gamma wave power values. A total of 4 min of data were calculated. Periodogram method is a method to estimate the power spectral density directly by Fourier transform of the sampled data *X*(*n*) of the signal. It is assumed that the finite length random signal sequence is *x*(*n*). Its Fourier transform and power spectral density have the following relationship:

(1) $${\tilde{S}}_{x}\,\left(f\right)=\frac{1}{N}\,{\left|x\,\left(f\right)\right|}^{2}$$

where *N* is the length of the random signal sequence *x*(*n*). At discrete frequency points *f* = *k*Δ*f*. There are:

(2) $${\tilde{S}}_{x}\,\left(k\right)=\frac{1}{N}\,{\left|X\,\left(k\right)\right|}^{2}=\frac{1}{N}\,{\left|F\,F\,T\,\left[x\,\left(n\right)\right]\right|}^{2}\,k=0,1\,\cdots ,N-1$$

where, FFT[*x*(*n*)] is Fast Fourier Transform of the sequence *x*(*n*). Because the period of FFT[*x*(*n*)] is *N*, the power spectrum estimation obtained took *N* as the period.

Finally, the average power of each electrode was calculated. For statistical analysis, 16 electrodes were selected to be divided into the following 10 brain regions: right occiput (O2), left occiput (O1), right parietal (P8, P4), left parietal (P7, P3), right central (C4), left center (C3), right frontal lobe (F8, F4, and Fp2), left frontal lobe (F7, F3, and Fp1), right temporal lobe (T8), and left temporal lobe (T7). Under each zone, the power values of the constituent electrodes are averaged, and the process is repeated for the alpha, theta, and gamma frequency bands.

## Experimental Results

To verify the impact of mindfulness adjustment on the EEG signals of the subjects, we conducted ANOVA using Statistical Package for the Social Sciences (SPSS) software (Wang et al., 2015). ANOVA (Harne and Hiwale, 2018) included the influence of mindfulness adjustment on frequency bands and the influence of different frequency bands in different brain regions. In neurofeedback, the changes in alpha, theta, and gamma waves are usually used as evaluation indicators, and corresponding improvement and treatment are executed by strengthening these waves. These are rhythmic waveforms produced by the brain during some activities. The characteristics of alpha wave are: it is easy to observe when people are in a relaxed, calm but awake state; Theta wave is characterized by its low frequency when people are sleepy; Gamma wave is characterized by: when the brain is engaged in a cognitive task, it connects neurons that have not been connected before to create a new working loop – popular understanding is that when creative thinking, creativity, and ideas suddenly appear at this time, gamma waves can be observed. Furthermore, related research shows that the main EEG indicators that are sensitive to mindfulness are alpha, theta, and gamma waves. Combining with previous research, the EEG indicators we chose while performing ANOVA were alpha, theta, and gamma waves.

**FIGURE 4:**
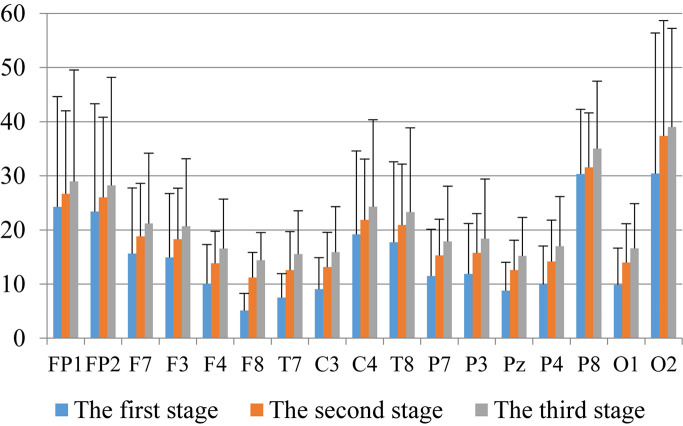
The characteristics of alpha wave power changes in whole brain regions of anxious subjects in the three experimental stages.

**FIGURE 5:**
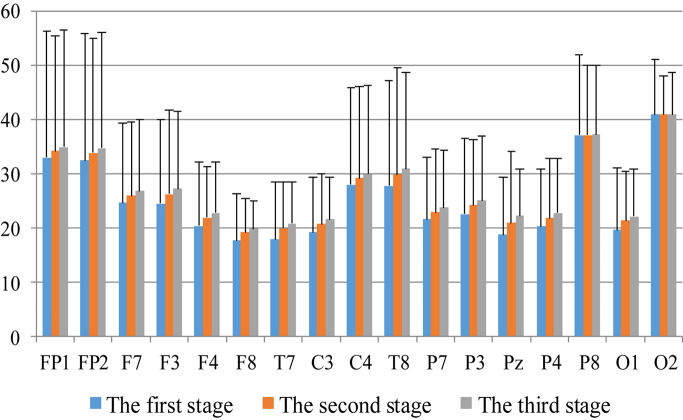
The characteristics of theta wave power changes in whole brain regions of healthy subjects in the three experimental stages.

**FIGURE 6:**
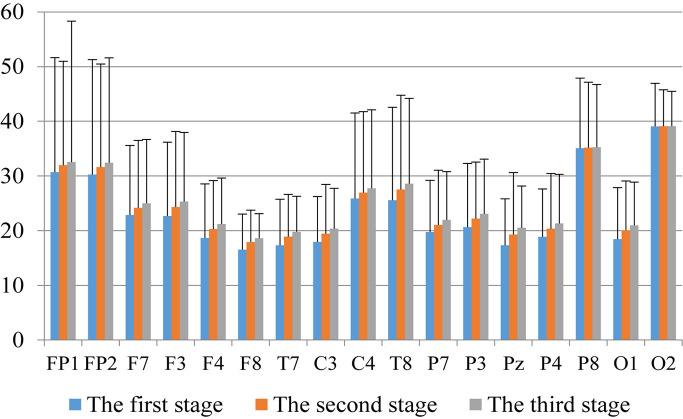
The characteristics of alpha wave power changes in whole brain regions of healthy subjects in the three experimental stages.

**FIGURE 7:**
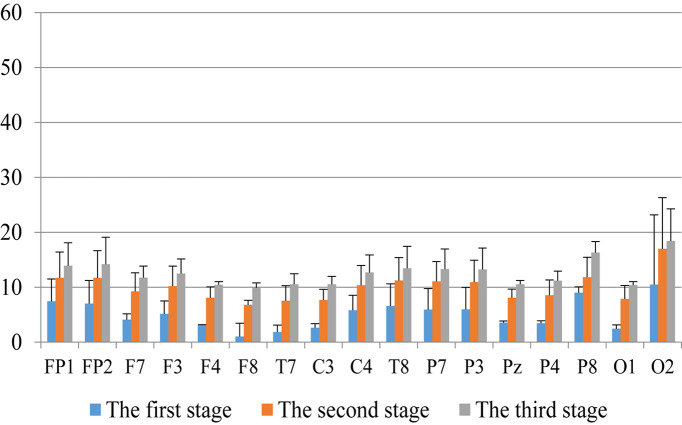
The characteristics of gamma wave power changes in whole brain regions of anxious subjects in the three experimental stages.

**FIGURE 8:**
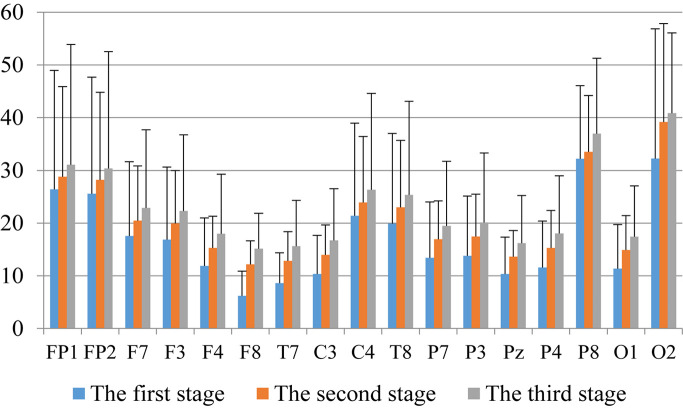
The characteristics of theta wave power changes in whole brain regions of anxious subjects in the three experimental stages.

**FIGURE 9:**
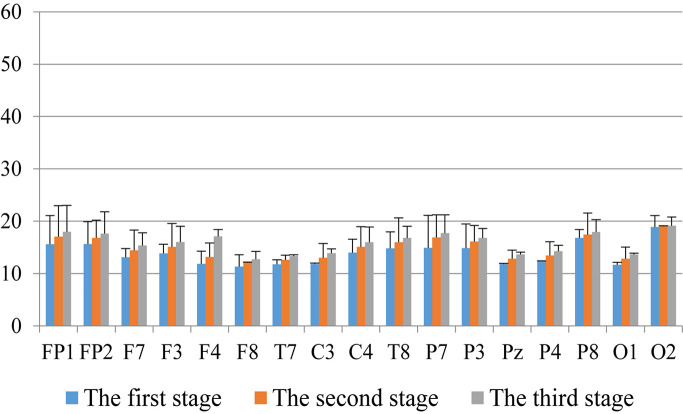
The characteristics of gamma wave power changes in whole brain area of healthy subjects in the three experimental stages.

First, we analyzed the change characteristics of the average power values of the alpha, theta, and gamma waves in the three stages of the experiment for anxious subjects and healthy subjects. It can be seen from Figures 4–6 that anxious subjects exhibited a characteristic that the power of alpha, theta, and gamma waves was generally very low prior to mindfulness adjustment, and the power gradually increased with this adjustment. This trend of change is consistent on each electrode, and the magnitude of the change is obvious. Mindfulness adjustment activates higher alpha, theta, and gamma waves. For healthy subjects, although the alpha wave also changed during the three stages of the experiment, this change is not evident in Figure 7, and not all electrodes show the same changes as the anxious subjects. For P8 and O2, the electrode changes can be seen as flat. Figures 8, 9 also show similar features to Figure 7.

Through the comparison between Figures 4–9, we can also see that the power value of the alpha wave of each lead of anxious subjects is lower than the power value of the alpha wave of healthy subjects. For theta and gamma waves, an analogous pattern was observed.

To assess the connection between the mindfulness regulation hemisphere, region, and condition, the wave bands were divided into hemispheres (left and right), regions (frontal, central, parietal, occipital, and temporal lobe), and condition (before and after mindfulness adjustment) as factors for three-way repeated ANOVA.

In Tables 1, 2, alpha, theta, and gamma waves were analyzed under anxiety and health conditions experimental results. Under the main effect, the number of treatment groups (*k* = 2), the number of samples (*n* = 390), the degree of freedom between groups was *k* − 1, and the degree of freedom within each group was *n* − *k*. In Table 1, *F*(1,338) = 127.65, which means that the degree of freedom of components is 1. The degree of freedom within the group is 338, and *P* = 2.50 ×  10^−25^ 0.05, which means that the difference between groups is significant. In condition × region interaction effect, it is divided into regions (frontal lobe, central lobe, parietal lobe, occipital lobe, and temporal lobe), number of treatment groups (*k* = 5), number of samples (*n* = 390), degree of freedom between groups is *k* − 1, degree of freedom within group is *n*(*k* − 1), *F*(4,339) = 20.52 in Table 1, indicating that the degree of freedom of component is 1, and degree of freedom within group is 339. *P* = 4.55 × 10^− 15^ 0.05, indicating a significant difference between groups. In condition×hemispheric interaction effect, it is divided into hemispheres (left and right), number of processing groups (*k* = 2), number of samples (*n* = 390), degree of freedom between groups is *k* − 1, and degree of freedom within group is *n* (*k* − 1). In Table 1, *F*(1,339) = 14.62, indicating that the degree of freedom between groups is 1, and the degree of freedom within group is 339. Similarly, *P* < 0.05, i.e., the difference between groups was significant, which was statistically significant.

**TABLE 1:** The average power variance analysis results of the two hemispheres, five regions, and two conditions in the alpha, theta, and gamma bands for anxious subjects before and after the experiment.

**Frequency band**	**Condition main effect**	**Condition × region interaction effect**	**Condition × hemisphere interaction effect**	**Condition × region × hemisphere interaction effect**
Alpha	*F*(1,338) = 127.65 *P* = 2.5010^− 25^	*F*(4,339) = 20.52 *P* = 4.55×10^− 15^	*F*(1,339) = 14.62 *P* = 1.50×10^− 4^	*F*(4,339) = 18.42 *P* = 1.21×10^− 13^
Theta	*F*(1,338) = 110.84 *P* = 1.31×10^− 22^	*F*(4,339) = 19.87 *P* = 1.26×10^− 14^	*F*(1,339) = 16.86 *P* = 5.10×10^− 5^	*F*(4,339) = 18.75 *P* = 7.20×10^− 14^
Gamma	*F*(1,338) = 633.73 *P* = 1.66×10^− 79^	*F*(4,339) = 6.51 *P* = 4.70×10^− 5^	*F*(1,339) = 3.74 *P* = 0.0439	*F*(4,339) = 5.65 *P* = 2.08×10^− 4^

*Significant (*P* < 0.0439).*

**TABLE 2:** The average power variance analysis results of the two hemispheres, five regions, and two conditions in the alpha, theta, and gamma bands for healthy subjects before and after the experiment.

**Frequency band**	**Condition main effect**	**Condition × region interaction effect**	**Condition × hemisphere interaction effect**	**Condition × region × hemisphere interaction effect**
Alpha	*F*(1,338) = 9.93 *P* = 0.0017	*F*(4,339) = 37.97 *P* = 5.33×10^− 26^	*F*(1,339) = 50.00 *P* = 9.69×10^− 12^	*F*(4,339) = 48.24 *P* = 9.89×10^− 32^
Theta	*F*(1,338) = 9.78 *P* = 0.0019	*F*(4,339) = 43.24 *P* = 4.25×10^− 29^	*F*(1,339) = 56.34 *P* = 6.08×10^− 13^	*F*(4,339) = 18.42 *P* = 6.86×10^− 25^
Gamma	*F*(1,338) = 77.13 *P* = 8.13× 10^− 17^	*F*(4,339) = 41.26 *P* = 6.95×10^− 28^	*F*(1,339) = 113.49 *P* = 6.95×10^− 23^	*F*(4,339) = 36.62 *P* = 3.31×10^− 25^

*Significant (*P* < 0.0017).*

The repeated ANOVA results in Tables 1, 2 indicated that both healthy and anxious subjects have significant main effects under the conditions of alpha, theta, and gamma zones. However, this main effect is more prominent in the latter. We can thus say that the analysis of our experimental results shows that neurofeedback can alleviate the anxiety of the subjects, and the alleviating effect on anxious subjects is evidently stronger than that on healthy subjects.

The interaction effects (hemisphere × region × condition, region × condition, and hemisphere × condition) are also obvious for alpha, theta, and gamma bands, and this significant effect is more intense in healthy subjects. To analyze the interaction between hemispheres, regions, and conditions in more detail, we performed a paired *t*-test on the alpha, theta, and gamma power before and after each region condition.

We made a significant analysis of the changes in the average power of alpha, theta, and gamma waves of EEG before and after the mindfulness adjustment of anxious subjects and healthy subjects in five brain regions. It can be seen from Tables 3–8 that regardless of subject, the average power of alpha, theta, and gamma waves in the five brain regions show very significant changes before and after mindfulness. In addition, in healthy subjects, there were observed higher significant changes than anxious subjects. It can be concluded that mindfulness adjustment made the brain’s electrical alpha, theta, and gamma waves of different subjects become more active, and this change was more significant among healthy subjects.

**TABLE 3:** The results of paired *t*-test for average alpha power before and after each regional condition for anxious subjects.

**Hemisphere**	**Region**	**Conditions**	**Paired differences**				
			**Mean ±** **SD**	**SE mean**	**95% confidence interval of the difference**		
					**Lower**	**Upper**	***P***
Left	Occipital	Before–after	−7.69 ± 3.09	0.75	–9.28	–6.10	1.94×10^− 8^
	Parietal	Before–after	−7.57 ± 3.22	0.78	–9.22	–5.91	4.28×10^− 8^
	Central	Before–after	−5.77 ± 4.05	0.98	–7.85	–3.69	2.30×10^− 5^
	Frontal	Before–after	−6.40 ± 3.65	0.88	–8.28	–4.52	2.00×10^− 6^
	Temporal	Before–after	−8.89 ± 2.90	0.70	–10.38	–7.39	9.83×10^− 10^
Right	Occipital	Before–after	−26.51 ± 14.09	3.41	–33.75	–19.26	8.23×10^− 7^
	Parietal	Before–after	−6.41 ± 1.65	0.40	–7.26	–5.56	2.91×10^− 11^
	Central	Before–after	−5.93 ± 4.13	1.00	–8.10	–3.85	2.00×10^− 5^
	Frontal	Before–after	−7.90 ± 3.25	0.78	–9.58	–6.23	2.64×10^− 8^
	Temporal	Before–after	−6.83 ± 3.62	0.87	–8.69	–4.96	2.12×10^− 7^

**TABLE 4:** The results of paired *t*-test for average theta power before and after each regional condition for anxious subjects.

**Hemisphere**	**Region**	**Conditions**	**Paired differences**				
			**Mean ±** **SD**	**SE mean**	**95% confidence interval of the difference**		
					**Lower**	**Upper**	***P***
Left	Occipital	Before–after	−7.15 ± 3.32	0.80	–8.86	–5.45	1.37×10^− 7^
	Parietal	Before–after	−7.38 ± 3.37	0.81	–9.12	–5.65	1.11×10^− 7^
	Central	Before–after	−5.75 ± 4.08	0.99	–7.85	–3.65	2.70×10^−5^
	Frontal	Before–after	−6.24 ± 3.79	0.92	–8.19	–4.29	4.00×10^− 6^
	Temporal	Before–after	−8.03 ± 2.94	0.71	–9.54	–6.52	5.11×10^− 9^
Right	Occipital	Before–after	−27.09 ± 14.45	3.50	–34.53	–19.66	8.64×10^− 7^
	Parietal	Before–after	−6.19 ± 1.75	0.42	–7.09	–5.28	1.20×10^− 10^
	Central	Before–after	−5.97 ± 4.21	1.02	–8.14	–3.81	2.40×10^− 5^
	Frontal	Before–after	−7.78 ± 3.47	0.84	–9.57	–6.00	8.15×10^− 8^
	Temporal	Before–after	−6.75 ± 3.74	0.90	–8.67	–4.82	1.00×10^− 6^

**TABLE 5:** The results of paired *t*-test for average gamma power before and after each regional condition for anxious subjects.

**Hemisphere**	**Region**	**Condition**	**Paired differences**				
			**Mean±SD**	**SE mean**	**95% confidence interval of the difference**		
					**Lower**	**Upper**	***P***
Left	Occipital	Before–after	−8.56 ± 2.16	0.52	–9.68	–7.45	2.11×10^− 11^
	Parietal	Before–after	−8.46 ± 3.13	0.76	–10.07	–6.85	6.05×10^− 9^
	Central	Before–after	−7.19 ± 2.55	0.62	–8.50	–5.87	3.39×10^− 9^
	Frontal	Before–after	−7.86 ± 2.42	0.58	–9.10	–6.61	4.21×10^− 10^
	Temporal	Before–after	−9.41 ± 2.34	0.56	–10.62	–8.20	1.77×10^− 11^
Right	Occipital	Before–after	−14.80 ± 7.35	1.78	–18.58	–11.02	3.41×10^− 7^
	Parietal	Before–after	−7.89 ± 1.48	0.36	–8.66	–7.13	2.39×10^− 13^
	Central	Before–after	−8.01 ± 2.68	0.65	–9.39	–6.63	1.40×10^− 9^
	Frontal	Before–after	−8.46 ± 2.25	0.54	–9.62	–7.30	4.69×10^− 11^
	Temporal	Before–after	−7.82 ± 2.84	0.68	–9.28	–6.36	4.52×10^− 9^

**TABLE 6:** The results of paired *t*-test for average alpha power before and after each regional condition for healthy subjects.

**Hemisphere**	**Region**	**Conditions**	**Paired differences**				
			**Mean±SD**	**SE mean**	**95% confidence interval of the difference**		
					**Lower**	**Upper**	***P***
Left	Occipital	Before–after	−2.54 ± 0.33	0.08	–2.71	–2.36	1.08×10^− 15^
	Parietal	Before–after	−2.35 ± 0.27	0.06	–2.49	–2.21	1.25×10^− 16^
	Central	Before–after	−2.06 ± 0.28	0.06	–2.21	–1.92	1.51×10^− 15^
	Frontal	Before–after	−2.29 ± 0.26	0.06	–2.43	–2.15	1.36×10^− 16^
	Temporal	Before–after	−2.48 ± 0.35	0.08	–2.66	–2.29	3.53×10^− 15^
Right	Occipital	Before–after	−0.01 ± 0.001	0.0003	–0.02	–0.01	4.07×10^− 20^
	Parietal	Before–after	−1.32 ± 0.09	0.02	–1.37	–1.27	7.60×10^− 20^
	Central	Before–after	−2.22 ± 0.34	0.08	–2.39	–2.04	9.19×10^− 15^
	Frontal	Before–after	−2.30 ± 0.31	0.07	–2.47	–2.14	1.86×10^− 15^
	Temporal	Before–after	−3.04 ± 0.39	0.09	–3.24	–2.83	5.81×10^− 16^

**TABLE 7:** The results of paired *t*-test for average theta power before and after each regional condition for healthy subjects.

**Hemisphere**	**Region**	**Conditions**	**Paired differences**				
			**Mean±SD**	**SE mean**	**95% confidence interval of the difference**		
					**Lower**	**Upper**	***P***
Left	Occipital	Before–after	−2.58 ± 0.47	0.11	–2.82	–2.33	1.84×10^− 13^
	Parietal	Before–after	−2.39 ± 0.32	0.07	–2.55	–2.22	1.13×10^− 16^
	Central	Before–after	−2.11 ± 0.29	0.07	–2.26	–1.96	1.85×10^− 15^
	Frontal	Before–after	−2.32 ± 0.29	0.07	–2.47	–2.17	5.24×10^− 16^
	Temporal	Before–after	−2.98 ± 0.58	0.14	–3.28	–2.68	4.03×10^− 13^
Right	Occipital	Before–after	−0.01 ± 0.001	0.0004	–0.01	–0.01	3.18×10^− 17^
	Parietal	Before–after	−1.31 ± 0.12	0.03	–1.38	–1.25	6.14×10^− 18^
	Central	Before–after	−2.26 ± 0.35	0.08	–2.44	–2.08	1.13×10^− 14^
	Frontal	Before–after	−2.34 ± 0.38	0.09	–2.54	–2.14	3.38×10^− 14^
	Temporal	Before–after	−3.15 ± 0.41	0.10	–3.36	–2.29	8.00×10^− 16^

**TABLE 8:** The results of paired *t*-test for average gamma power before and after each regional condition for healthy subjects.

**Hemisphere**	**Region**	**Conditions**	**Paired differences**				
			**Mean±SD**	**SE mean**	**95% confidence interval of the difference**		
					**Lower**	**Upper**	***P***
Left	Occipital	Before–after	−1.99 ± 0.07	0.02	–2.03	–1.96	8.98×10^− 25^
	Parietal	Before–after	−2.39 ± 0.33	0.08	–2.57	–2.22	1.94×10^− 15^
	Central	Before–after	−2.40 ± 0.22	0.05	–2.51	–2.29	3.40×10^− 18^
	Frontal	Before–after	−2.26 ± 0.13	0.03	–2.33	–2.19	4.61×10^− 21^
	Temporal	Before–after	−1.61 ± 0.05	0.01	–1.63	–1.58	1.39×10^− 25^
Right	Occipital	Before–after	−0.28 ± 0.003	0.0008	–0.28	–0.27	6.31×10^− 32^
	Parietal	Before–after	−1.48 ± 0.02	0.007	–1.50	–1.47	5.71×10^− 29^
	Central	Before–after	−1.97 ± 0.12	0.03	–2.04	–1.91	1.26×10^− 20^
	Frontal	Before–after	−1.86 ± 0.09	0.02	–1.91	–1.82	2.35×10^− 22^
	Temporal	Before–after	−2.00 ± 0.09	0.02	–2.05	–1.95	1.31×10^− 22^

As shown in Figures 4–6 for anxious subjects, the average power of the EEG biomarkers alpha, theta, and gamma waves of the subjects before and after the neurofeedback mindfulness adjustment increased significantly, indicating that our experiment activated higher alpha, theta, and gamma activities of the subjects, and this change is significant in each brain area. Tables 1, 3–5 can illustrate this significance. Healthy subjects did not manifest any tendency toward anxiety. They appeared to be in control throughout the experiment. Figures 7–9 depict that although healthy subjects were tested before and after neurofeedback mindfulness adjustment, the changes in average power of the EEG biomarkers alpha, theta, and gamma waves are not as large as that of anxious subjects. However, they also show a trend of power increase, indicating that our experiment also provided a certain relief to healthy subjects’ mood, the role of auxiliary regulation. Tables 2, 6–8 show that this subtle change is also significant.

## Discussion

In previous studies, the assessment of anxiety relief chiefly relied on some anxiety scales (Zafeiri et al., 2019). Whether it is before or after adjustment, anxiety is relieved and is too one-sided based on the scores of a single scale. Subjects are often not aware of their own situation. For some subjects, the anxiety scale is obscure and difficult to understand, and they appear anxious or even fidgeting during evaluation. It is unclear what such evaluation results can indicate. Although the participant’s scale score can explain the relief of anxiety symptoms, the subjectivity of the scale evaluation is still too strong, and thus it needs to be evaluated from an objective perspective to reflect the true state of the participant, and the scale evaluation can be used as an auxiliary evaluation means.

This study was aimed to assess the relieving effect of neurofeedback mindfulness regulation on subjects’ anxiety, and to objectively evaluate this relieving effect through neurophysiological signals. The effect on anxiety relief was judged by analyzing the change characteristics of the average power of alpha, theta, and gamma waves of the brain’s electrical signal 4 min before and after the subject’s neurofeedback mindfulness adjustment.

The alpha, theta, and gamma band power were evaluated before and after neurofeedback mindfulness regulation. For anxiety disorder patients, the average alpha, theta, and gamma power is generally very low before the regulation of mindfulness, and gradually increases with the regulation of mindfulness. For healthy subjects, it can be observed that the power of alpha, theta, and gamma bands increased not obviously, compared with anxiety disorder patients. Additionally, the power of each band of patients was lower than healthy subjects. The statics analysis showed the significant effect of brain activities after neurofeedback.

In the past, neurofeedback has been used to regulate brain activity and reduce alpha asymmetry to improve anxiety in patients with depression and anxiety. However, different types of intervention are required for different patients with different duration of training, and the sample size is too small. The data used in the evaluation process is relatively simple, and there are incorrect experimental data, which will affect the results of the experiment. For example, Dias divided 87 patients with major depressive disorder and anxiety into alpha-asymmetry neurofeedback (ALAY), high-beta down-training, or control groups. Both neurofeedback groups received 10 sessions of neurofeedback (Dias and van Deusen, 2011) and had reduced symptoms of depression and anxiety. Compared with the other groups, the BETA group was more effective at reducing the high beta power in the parietal cortex, but it may take more than 10 repetitions of training to reach the neurofeedback goal. In addition, Cheon et al. (2015) modified the 8-week ALAY neurofeedback regimen to increase the beta power of the left frontal cortex (F3) and decrease the alpha power, while increasing the theta (alpha/theta ratio) of the parietal cortex in depressed patients. The results demonstrated that within 8 weeks, depression and anxiety symptoms were significantly reduced, as was the clinical severity of psychiatric symptoms. The 15 patients in the feedback group were given the neurofeedback training of alpha enhancement, theta enhancement, and beta3 reduction, three times a week for 4 weeks. The fake feedback group did not give real feedback, but simply played back previous training data from other people for the same amount of time as the neurofeedback group. Patients in both groups were treated with the same drug (duloxetine hydrochloride 60 mg once daily). Results After training, the alpha and theta amplitude of the feedback group were significantly higher than that of the false feedback group, and the beta3 amplitude had a downward trend; however, there was no statistical difference (*P*-value was 0.004, 0.038, and 0.818, alpha, theta, and beta3, respectively). However, the feedback group had the function of helping to improve the anxiety of patients with generalized anxiety disorder.

This experiment has made some progress in the evaluation of anxiety state, which is only a small step forward, and there is still a lot of room for improvement in the accuracy of the evaluation. Due to the limited research time and small sample size, there may be many methods that can be applied to the assessment of anxiety state, and the future research prospects are broad. In the near future, we will need to optimize our experiments to improve the relief level of anxiety symptoms of anxiety subjects to the level of healthy subjects.

To sum up, the nervous feedback can effectively control the brain wave patterns and achieve cure, and possesses the advantages of non-invasive, less adverse reaction, the characteristics of being simple, safe, and convenient (Vernon et al., 2003). Through the analysis of neurophysiological signals, it can be concluded that our experiment can alleviate the anxiety symptoms of the subjects. In the current period of new crown epidemic most people suffer from anxiety, whether it is healthy people or patients with anxiety disorders. We hope that our experiment can provide people with relief from their anxiety.

## Data Availability Statement

The datasets presented in this article are not readily available because the experiment data is not available online for further research, but available on reasonable request according to the policy of Tianjin University, Capital Medical University, and Tianjin University of Technology. Requests to access the datasets should be directed to CC, cccovb@hotmail.com.

## Ethics Statement

All procedures performed in studies involving human participants were in accordance with the ethical standards of Ethics Committee of Beijing Anding Hospital, Capital Medical University (ZYLX201607). This ethics committee’s responsibilities, composition, function, operations, and records are fully compliant with ICH-GCP, and related regulation and law of China. The patients/participants provided their written informed consent to participate in this study.

## Author Contributions

CC, XX, AB, and CW recorded the original experiment data, analyzed the experiment data, and wrote parts of the manuscript. SS and XZ completed the ethic files of this experiment. LL, XW, WY, and PL wrote parts of the manuscript. CW, CC, and DM designed the experiment and revised the manuscript. All authors contributed to the article and approved the submitted version.

## Conflict of Interest

The authors declare that the research was conducted in the absence of any commercial or financial relationships that could be construed as a potential conflict of interest.

## Publisher’s Note

All claims expressed in this article are solely those of the authors and do not necessarily represent those of their affiliated organizations, or those of the publisher, the editors and the reviewers. Any product that may be evaluated in this article, or claim that may be made by its manufacturer, is not guaranteed or endorsed by the publisher.
